# New, goal-directed approach to renal replacement therapy improves acute kidney injury treatment after cardiac surgery

**DOI:** 10.1186/1749-8090-9-103

**Published:** 2014-06-18

**Authors:** Jiarui Xu, Xiaoqiang Ding, Yi Fang, Bo Shen, Zhonghua Liu, Jianzhou Zou, Lan Liu, Chunsheng Wang, Jie Teng

**Affiliations:** 1 Department of Nephrology, Zhongshan Hospital, Shanghai Medical College, Fudan University, No 180 Fenglin Road, Shanghai 200032, China; 2 Shanghai Institute of Kidney and Dialysis, No 180 Fenglin Road, Shanghai 200032, China; 3 Department of Cardiovascular Surgery, Zhongshan Hospital, Shanghai Medical College, Fudan University, No 180 Fenglin Road, Shanghai 200032, China

**Keywords:** Cardiac surgery, Acute kidney injury, Goal-directed renal replacement therapy, Daily hemofiltration

## Abstract

**Aim:**

The aim of this study was to compare the efficacies of goal-directed renal replacement therapy (GDRRT) and daily hemofiltration (DHF) for treating acute kidney injury (AKI) patients after cardiac surgery.

**Methods:**

In our retrospective study, we included 140 cardiac surgery AKI patients who were treated with renal replacement therapy (RRT) from 2002 to 2010. Two patient groups, which comprised 70 patients who received DHF from January 2002 to September 2008 and 70 patients treated with GDRRT from October 2009 to September 2010 were pair-wise compared regarding clinical outcomes, as well as the incidence of adverse events.

**Results:**

In-hospital and 30-day mortality rates were 45.7% and 41.4% in the GDRRT and 48.6% and 54.3% in the DHF group, respectively, but without statistically significant differences. GDRRT patients needed statistically significantly shorter hospital and intensive care unit (ICU) stays, less frequent RRT, and shorter RRT sessions, whereas, of 11 analyzed renal outcome parameters, 6 values, including percentage of complete renal recovery and time for complete renal recovery, were significantly superior in the GDRRT group at the time of discharge. There was no significant difference in the incidence of adverse events within the initial 72 treatment hours between the 2 groups. Hospitalization expenses were less in GDRRT group than in DHF group.

**Conclusion:**

The GDRRT approach is superior to DHF for improving renal outcome, as well as reducing the time and cost of RRT therapy, for cardiac surgery AKI patients.

## Background

Cardiac surgery is a major cause of AKI in critically ill patients [1,2], and mortality among cardiopulmonary bypass patients who need RRT after cardiac surgery is reported to be 60-70% [3]. Although RRT is important in saving critically injured AKI patients, there is no consensus regarding the optimal dose or form. There is insufficient evidence to show that continuous renal replacement therapy (CRRT) is better than intermittent renal replacement therapy (IRRT) [4,5], and the efficacy of hybrid patterns, such as sustained low-efficiency dialysis (SLED), has not yet been evaluated [6]. It is also unclear what the optimal dose is, although it is argued that, for critically injured AKI patients, the dose should be at least 35 ml/kg/h of hemofiltration (HF) and an spKt/V value of 1.4 for hemodialysis (HD) [7]. However, recent results from the Acute Renal Failure Trial Network (ATN) study and the RENAL or IVOIRE trials did not suggest the superiority of high-dose intensive RRT [8,9]. Another issue is the optimal intervention time, and most clinicians believe that early RRT can improve outcomes, but there is no uniform standard as to when RRT should be initiated [10-12]. The idea of a goal-directed therapy (GDT) for critically ill patients was first raised in 1973 by Shoemaker [13], and hemodynamic GDT after surgery has been acknowledged as an effective measure against postoperative hospital-acquired infections [14]. In addition, various GDTs have been developed to achieve hemodynamic stability during and after cardiac [15,16] and non-cardiac surgery [17], and Goldstein et al. have proposed an early goal-directed fluid therapy (EGDT) for fluid management of critically ill AKI patients [18]. The concept of a GDRRT was first proposed by Mehta et al. [19], but up to now, no detailed studies have been published about GDRRT. We have been using GDRRT since 2008, in which RRT goals were set up first, followed by determinations of ultrafiltration rates, dialysate compositions, and goal-directed anticoagulant therapies. GDRRT is supposed to be beneficial not only by providing extra treatments when they are specifically indicated, but also by allowing the earlier and guided use of fluids and preventing the delivery of unnecessary treatments or fluid removal when specific objectives have already been achieved. In this retrospective analysis, we compared the treatment outcomes of GDRRT and DHF in patients with AKI after cardiac surgery.

## Methods

### Patients

This retrospective, observational study was performed in a cohort of 140 adult patients who underwent cardiac surgery in our hospital from January 2002 to September 2010. The study included adult patients with AKI who required RRT after open heart surgery and survived longer than 72 h after surgery. Patients were excluded if they were under the age of 18, received cardiac transplantation surgery, had end-stage renal disease (ESRD) before surgery, or received preoperative RRT due to any reasons. The DHF group included 70 patients who received DHF between January 2002 and September 2008, and the GDRRT group was comprised of 70 patients who were treated with GDRRT between October 2009 and September 2010. We used pair-wise comparisons between the 2 groups regarding clinical outcomes, as well as the incidence of adverse events. The study was approved by the Institutional Review Board of the Shanghai Zhongshan Hospital, which waived the requirement for informed consent for the retrospective review of RRT interventions after cardiac surgery. In our hospital, we practiced only DHF as a therapeutic strategy for treating AKI following cardiac surgery until 2008, and GDRRT was first introduced in October 2008. We used paired analysis in order to reduce the bias created by the different time periods. We collected data for a paired analysis from 70 patients who received DHF between January 2002 and September 2008, and from 70 patients who received GDRRT between October 2009 and September 2010. The main criteria for the paired analysis were equal type of surgery and severity of illness, according to acute physiology and chronic health evaluations (APACHE) II [20] and sepsis-related organ failure assessment (SOFA) scores [21].

### Data collection

In addition to hemodynamic and laboratory data, baseline vital signs were also recorded in the first day of ICU admission and then daily after nephrology consultation. In addition, APACHE, II as well as SOFA scores and EuroScore values [22], were computed within the first 24 h after admission to the ICU, in order to assess the severity of the illness. AKI was defined as elevation of serum creatinine (SCr) ≥ 26.4 μmol/L or 50% higher than the baseline within 48 h, or urine output < 0.5 ml/kg/h for more than 6 h, and staged according to the AKI Network classification [23]. Sepsis was defined according to the American College of Chest Physicians/Society of Critical Care Medicine (ACCP/SCCM) Consensus Conference Committee [24]. Recovery of kidney function was considered to be complete if the SCr was less than 44 μmol/L above the baseline value or to be partial if the SCr remained higher than 44 μmol/L above the baseline value but the patient was not dialysis-dependent [9]. In-hospital mortality rates inside and outside the ICU within 30 days were the primary end-points of the trial. Secondary end-points were ICU and hospital days, mechanical ventilation days, hospitalization expenses, hemodynamic stability, control of azotemia and volume overload, as well as renal function recovery.

### RRT methods

#### ***DHF***

The form, frequency, and time of each session, as well as the dose/ultrafiltration rate of DHF were fixed, assuming that patients were in a stable state at the time of DHF initiation and that their status did not change substantially. The indications for RRT initiation were as follows: (1) Serum K^+^ ≥ 6.0 mmol/L and/or electrocardiogram abnormalities; (2) Arterial blood pH ≤ 7.15; (3) Urine output < 200 mL/12 h when the first dose of furosemide was up to 80 mg or the highest dose was up to 240 mg, or anuria; (4) Rapidly rising blood urea nitrogen (BUN) or SCr; (5) Refractory fluid overload with pulmonary edema; (6) Severe sepsis with septic shock. HF was performed with Baxter BM25 or Aquarius CRRT equipment using FILTRAL 20 (AN 69, 2.05 m^2^ surface, GAMBRO, Sweden). We used our hospital formula as a replacement fluid, and the ratio of pre- and post-dilution was two-thirds, with an ultrafiltration rate of 60-70 ml/kg/h. Vascular access was obtained with a dual-lumen HD catheter inserted into the internal jugular or femoral vein by the standard Seldinger technique. Either a loading dose of 1500 IU heparin followed by an individual patient-adjusted anticoagulation regimen or no anticoagulation was used.

#### ***GDRRT***

GDRRT cannot be defined as a fixed RRT strategy, and it is basically composed of HD, HF, and hemodiafiltration (HDF). All of these techniques were used, in order to achieve specific goals, and various parameters were monitored. The indications for RRT initiation were the same as for DHF. The patients received a central venous catheter capable of measuring central venous pressure (CVP), and oxygen saturation data were fed into a computerized ECG monitor, which also monitored heart and breathing rates, as well as blood pressure. Fluid intake and output was recorded every day, and urine output was recorded every 6 h. Potential of hydrogen and arterial oxygen saturation were monitored by blood gas analysis at least every 4 h. Blood biochemistry and other laboratory data were tested at least once every day. The usual HD or HF lasted about 4 h per session and were performed every day or every other day, but for all patients, the form, frequency, and time of each session and the dose/ultrafiltration rate were not standardized. The procedures were all performed with Baxter Aquarius equipment, using highly permeable membranes made of polyether sulfone (1.7 m^2^ or 1.4 m^2^ surface area, GAMBRO, Sweden) starting the first session with a small surface area filter and increasing the filter size in further sessions. The vascular access and replacement fluid were the same as for DHF, and the usual HF ultrafiltration rate was 25-35 ml/kg/h. The usual blood flow ranged from 100 to 250 ml/min and the dialysate flow was 8-10 L/h for HD. In all GDRRT modalities, anticoagulation was performed with unfractionated heparin, low-molecular-weight heparin (LMWH), or no anticoagulation, according to the consulting nephrologists’ decisions.

#### ***GDRRT algorithm***

The goals of GDRRT are listed in Table 1, and they included immediate and ongoing goals. The immediate goals were correcting electrolyte disturbance and acidemia by providing bicarbonate or reducing hyperkalemia, controlling volume by removing fluid overload, and improving hemodynamic stability. Ongoing goals included maintenance of fluid balance, promoting renal recovery, weaning from vasopressors, maintenance of acid-base and electrolyte balances, and support of organ functions.

**Table 1:** The goals of GDRRT

Solute	BUN ≤ 30 mmol/L
Volume	Urine output ≥ 0.5 ml/kg/h, 24 h fluid output ≥ 24 h fluid intake in volume overload patients, controlled acute pulmonary edema, reduction of peripheral edema, hematocrit ≥ 30%
Electrolyte and pH	Electrolyte and acid-base parameters normal or near normal: 3.5 < potassium ≤ 5.5 mmol, 7.25 ≤ pH < 7.45
Hemodynamics	MAP ≥ 65 mmHg without vasoactive drugs, CVP ≥ 8–12 mmHg, SaO_2_ ≥ 93%

BUN = blood urea nitrogen, MAP = mean arterial pressure, CVP = central venous pressure, SaO2 = saturation level of oxygen in hemoglobin.

**Figure 1:**
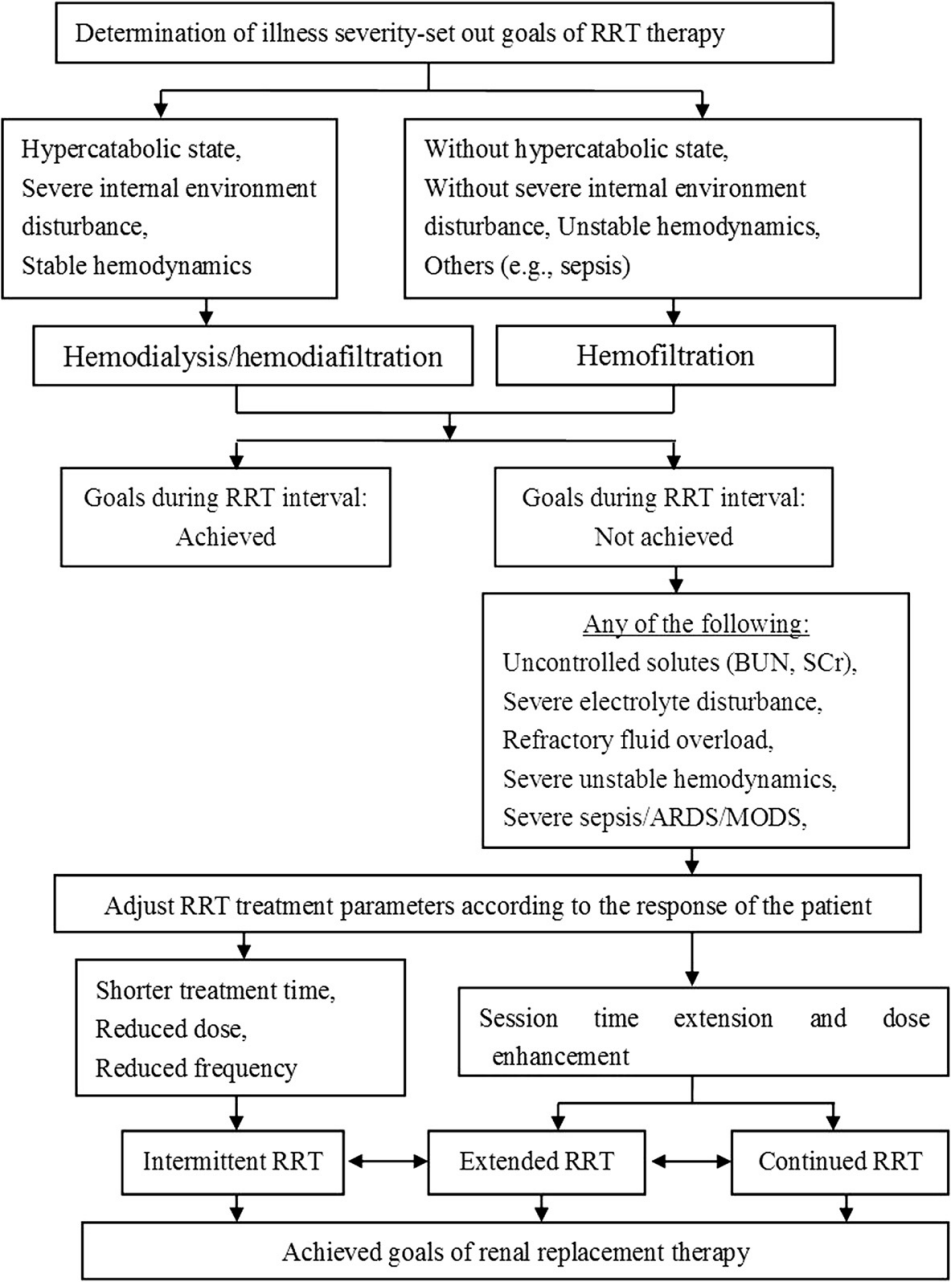
Scheme of the GDRRT algorithm.

The treatment protocol was as follows (see Figure 1). The GDRRT strategy was decided before treatment, based on the severity of the illness. Hemodynamically stable patients in hypercatabolic state or with severe internal environment disturbance (defined as having a SOFA cardiovascular score of 0 to 2) received HD or HDF, while patients without hypercatabolic state or severe internal environment disturbance or who were hemodynamically unstable (defined as having a SOFA cardiovascular score of 3 to 4) received HF. GDRRT was performed every day to achieve the goals listed in Table 1, and the frequency and duration of RRT sessions were determined depending on whether these goals were achieved. If the goals were not achieved (e.g., no solute control, severe electrolyte disturbance, refractory fluid overload, severe unstable hemodynamics), or in case of severe sepsis, acute respiratory distress syndrome (ARDS), or multiple organ dysfunction syndrome (MODS), the dose was increased and the usual HD and HF session times of about 4 h were extended to 8–10 h per session, or even 24 h if necessary. If the goals were achieved in the following days, the dose and frequency were reduced, and the time of each session was shortened. When patients receiving HD turned hemodynamically unstable (i.e., when the mean arterial pressure (MAP) was ≤ 65 mmHg, or the average cumulative daily variability of MAP was ≥ 30 mmHg) they were transitioned to HF, or continuous treatment was administered and vasopressors or vasodilators were given to maintain the MAP. The crossover from one to the other RRT modality was decided by nephrologists. The initial form of GDRRT included 57 usual HD and 13 HF sessions, and the main reason for conversion from HD to HF was hemodynamic instability. During the course of treatment, 10 patients received continued HD/HF because of sepsis or MODS, and only one patient received continued HDF for severe sepsis. The ultimate form of GDRRT was usual HD 53 times and HF 17 times.

### Statistical analyses

Statistical analyses were performed using the SPSS software, version 16.0. Measurement data were expressed as means ± SD or median and quartile **[**M (QR)**]**. Paired t-test was used for comparing baseline characteristics of the 2 groups, and independent t-test was used for comparing outcomes, while chi-squared test was used to analyze categorical data. Logistic regression analysis was used to correct for confounding factors. P < 0.05 was considered as statistically significant.

## Results

Of the 140 patients, 70 underwent GDRRT, while the other 70 underwent DHF, and treatments in the 2 groups included 21 valve surgeries, 13 coronary artery bypass grafts (CABG), 19 thoracic aortic surgeries, 7 congenital heart surgeries, and 10 combined surgeries. The body mass index (BMI) in the GDRRT group was significantly lower than in the DHF group (22.0 ± 3.6 vs. 23.8 ± 4.6, P = 0.025). Other baseline characteristics of the 2 groups were not significantly different (Table 2).

**Table 2:** Baseline characteristics in the GDRRT and DHF groups

	**GDRRT (n =70)**	**DHF (n = 70)**	**P value**
Male (n/%)	70 (64%)	70 (71%)	0.366
Age (y)	54 ± 15	55 ± 15	0.788
BMI (kg/m^2^)	22.0 ± 3.6	23.8 ± 4.6	0.025
DM (%)	16%	7%	0.118
HT (%)	33%	44%	0.165
NYHA III	44%	54%	0.119
IV	17%	28%	0.061
CPB duration (min)	116 ± 59	134 ± 52	0.065
Aortic clamping duration (min)	58 ± 38	57 ± 32	0.919
EuroSCORE			
Low risk (<3)	12 (17.1%)	11 (15.7%)	0.820
Medium risk (3 - 6)	28 (40%)	25 (35.7%)	0.601
High risk (>6)	30 (42.9%)	34 (48.6%)	0.497
Pre-op BUN (mmol/L)	9.4 ± 5.0	8.9 ± 6.7	0.632
Pre-op SCr (μmol/L)	112.1 ± 74.8	119.2 ± 101.7	0.639
Renal function before RRT			
BUN (mmol/L)	24.8 ± 15.2	27.3 ± 16.2	0.378
SCr (μmol/L)	334.5 ± 148.9	365.0 ± 156.3	0.250
K^+^ (mmol/L)	4.6 ± 1.2	4.5 ± 1.3	0.637
Oliguria (%)	48%	44%	0.683
AKIN before RRT (%)			
AKIN1	19%	12%	0.160
AKIN2	31%	25%	0.840
AKIN3	50%	63%	0.211
Time from ICU admission			
to RRT (d)	2 (1, 5)	2.5 (2, 5)	0.245
Sepsis (%)	18%	23%	0.381
Post-op APACHE II (24 h)	21.9 ± 5.4	22.8 ± 4.0	0.266
Post-op SOFA (24 h)			
Cardiovascular	2.8 ± 1.1	2.6 ± 1.0	0.262
Respiratory	2.4 ± 1.0	2.1 ± 1.5	0.164
Renal	1.8 ± 0.9	1.7 ± 1.2	0.587
Entire SOFA Score	10.8 ± 2.5	11.4 ± 2.8	0.183

NYHA: New York Heart Association; Pre-op BUN: preoperative blood urea nitrogen; Pre-op Scr: preoperative serum creatinine; Post-op APACHE II (24 h): postoperative acute physiology and chronic health evaluation within the first 24 h after surgery; Post-op SOFA (24 h): postoperative sepsis-related organ failure assessment within the first 24 h after surgery.

### Clinical outcomes

After being adjusted by logistic regression, the in-hospital mortality in the GDRRT and DHF groups were 45.7% and 48.6%, respectively (P = 0.734). The 30-day mortality was 41.4% and 54.3% in the GDRRT and the DHF groups, respectively. The median hours of ICU stay and mechanical ventilation days in the GDRRT group were significantly less than those in the DHF group (238.9 [119.9-483.8] vs. 360.0 [178.8-552.9] hours, P = 0.026) and 6 [3-15] vs. 9 [6-19] days, respectively, P = 0.046). There was no significant difference in median length of hospital stay between the 2 groups (25 [14-32] vs. 23 [13-36] days, P = 0.998). The frequency of RRT and the mean duration of RRT sessions were longer in the DHF group (2 [1-6] vs. 4 [2-9], P = 0.018 and 5 ± 1 vs. 6 ± 2 hours, P < 0.01). Hospitalization expenses were much higher in the DHF than in GDRRT group (2307.7 ± 430.8 vs. 1515.4 ± 461.5 dollars, P < 0.01) (Table 3).

**Table 3:** Clinical outcomes of AKI patients in the GDRRT and DHF groups

	**GDRRT (n = 70)**	**DHF (n = 70)**	**P value**
Hospital mortality (%)	45.7%	48.6%	0.734
30-d mortality (%)	41.4%	54.3%	0.128
Hospital stay (d)	25 (14, 32)	23 (13, 36)	0.998
ICU stay (h)	238.9 (119.9 - 483.8)	360.0 (178.8 - 552.9)	0.026
Mechanical ventilation days	6 (3 - 15)	9 ( 6 - 19)	0.046
Hospitalization expense (US$)	1515.4 ± 461.5	2307.7 ± 430.8	< 0.01
RRT frequency	2 (1 - 6)	4 (2- 9)	0.018
Duration of the RRT sessions			
First (h)	4 ± 1	5 ± 1	< 0.01
Average (h)	5 ± 1	6 ± 2	< 0.01

### Renal outcomes

The complete renal recovery rate was significantly higher in the GDRRT than in DHF group (37% vs. 19%, P = 0.016). The time for complete renal recovery was shorter in the GDRRT group (13 ± 9 vs. 22 ± 14 days, P = 0.042), whereas there were no significant differences in partial renal recovery rates in the 2 groups (9% vs. 15%, P = 0.409). Both maximum SCr and SCr at hospital discharge were higher in the DHF than in the GDRRT group (561.0 ± 239.2 vs. 441.7 ± 189.9 μmol/L, P < 0.001, and 377.2 ± 265.8 vs. 275.5 ± 164.3 μmol/L, P = 0.007). There were no significant differences in maximum BUN and BUN at hospital discharge between the 2 groups (45.5 ± 26.1 vs. 52.2 ± 23.5 mmol/L, P = 0.226; 34.2 ± 20.9 vs. 29.6 ± 17.7 mmol/L, P = 0.356). Urinary volume after 72 h treatment was much greater in the GDRRT than in DHF group (1590 [450-3285] vs. 370 [84, 1365] ml, P = 0.002, and the time to oliguria resolution was shorter in the GDRRT group (2.5 [1.0, 3.8] vs. 5 [2.5, 8.5] days, P = 0.033) (Table 4).

**Table 4:** Renal outcomes of AKI patients in the GDRRT and DHF groups

	**GDRRT (n = 70)**	**DHF (n = 70)**	***P *** **value**
Max BUN (mmol/L)	52.2 ± 23.5	45.5 ± 26.1	0.226
Max SCr **(**μmol/L)	441.7 ± 189.9	561.0 ± 239.2	< 0.001
BUN at discharge (mmol/L)	29.6 ± 17.7	34.2 ± 20.9	0.356
Scr at discharge (μmol/L)	275.5 ± 164.3	377.2 ± 265.8	0.007
K^+^ after RRT ((mmol/L)	4.5 ± 0.8	4.8 ± 0.5	0.008
Urinary volume after 72 h (ml)	1590 (450 - 3285)	370 (84- 1365)	0.002
Oliguria resolve time (d)	2.5 (1.0 - 3.8)	5 (2.5 - 8.5)	0.033
Complete renal recovery (%)	37%	19%	0.016
Time for complete renal recovery (d)	13 ± 9	22 ± 14	0.042
Partial renal recovery (%)	9%	15%	0.409
RRT independence at discharge (%)	42%	29%	0.111

Max BUN = maximum blood urea nitrogen, Max SCr = maximum serum creatinine.

### Hemodynamic parameters and safety of therapy

The incidence of tachycardia and blood coagulation was significantly higher in the DHF than in the GDRRT group (79% vs. 60%, P = 0.018; 37% vs. 21%, P = 0.041). There were no significant differences in MAP values and in the incidence of hypotension within the first 72 h of treatment between the 2 groups (82 ± 12 vs. 83 ± 15 mmHg, P = 0.664; 38% vs. 40%, P = 0.862). There was no significant difference in the daily ultrafiltration volume between the 2 groups (2112 ± 768 vs. 1925 ± 866 ml, P = 0.179). The major anticoagulant medication in the DHF group was LMH (60%), whereas in the GDRRT group, most patients (67%) received no anticoagulation medication (Table 5).

**Table 5:** Hemodynamic parameters and adverse events within the first 72 h of treatment

	**GDRRT (n = 70)**	**DHF (n = 70)**	***P *** **value**
Tachycardia (%)	60%	79%	0.018
MAP (mmHg)	82 ± 12	83 ± 15	0.664
Hypotension (%)	38%	40%	0.862
Blood coagulation (%)	21%	37%	0.041
Daily ultrafiltration volume (ml)	2112 ± 768	1925 ± 866	0.179
Anticoagulant therapies			
Unfractionated heparin	5%	20%	< 0.01
LMWH	28%	60%	< 0.01
No therapy	67%	20%	< 0.01
Dialysate + filtration rate (ml/kg/h)	24.2 ± 11.1	63.7 ± 12.6	

MAP = mean arterial pressure; LMWH = low-molecular-weight heparin.

## Discussion

Although high-dose HF can help alleviate systemic inflammatory responses, it also can be harmful, due to its non-selective filtration, which can lead to the loss of heat, nutrients, and drugs [25]. In our study, GDRRT was mostly composed of HD, which can correct azotemia and acid-base disorders. High-dose HF was employed only for patients with severe sepsis, in order to support the internal environment and extra-renal organs, and to control inflammation and achieve hemodynamic stability, thereby improving immune paralysis and ventilatory functions. However, the incidence of sepsis in our study was only 18% for the GDRRT group and 23% for the DHF patients - lower than the previously reported values of 62.4% [9] and 46.8% in RTT patients after surgery. It has been suggested that HD often results in hemodynamic instability, but in this study, hypotension occurred at similar rates during intermittent HD sessions in the 2 groups, and tachycardia occurred more frequently in the DHF group. This can be explained by the low ultrafiltrate rate, which maintained stable hemodynamics in the GDRRT group, because the HD dose in the DHF group was significantly higher than in the GDRRT group (63.7 vs. 24.2 ml/kg/h). These values were also similar to the previously published values of 57 ml/kg/h for IRRT and 42 ml/kg/h for continued veno-veno HF (CVVH) treatment for cardiac surgery AKI patients [26]. The lower ultrafiltrate rate also reduced the need for anticoagulant medication and led to a lower incidence of blood coagulation in the GDRRT group.

The absence of complete renal recovery from AKI is one of the major risk factors of ESRD [27]. Bagshaw et al. reported that, of 240 critically ill patients who required dialysis, 32% were on chronic renal replacement therapy at hospital discharge and 22% persisted on dialysis after 1 year. Others also suggested that one of the purposes of RRT in treating AKI should be the promotion of renal function recovery and independence from dialysis as soon as possible [28,29]. But the use of higher dialysis dose doesn’t appear to have a strong influence on renal recovery [9]. In our study, GDRRT led to significantly greater elimination of BUN and SCr, along with correction of hyperkalemia (Table 4). GDRRT was mainly composed of bedside HD, which may correct azotemia and electrolyte disorders more effectively than DHF. Because of the flexibility of GDRRT regarding dose and treatment time, urinary volume after 72 h of treatment was much higher and oliguria resolved much faster in the GDRRT group, which may have resulted in better renal outcome.

Even though treatment with GDRRT did not result in a survival advantage over DHF, the complete renal recovery rate was significantly higher in the GDRRT group compared with the DHF group (37% vs. 19%, P = 0.016) and less time was necessary for renal recovery (13.9 vs. 22.14 days, P = 0.042). The lack of a statistically significant difference in hospital mortality between the 2 groups might have been due to the small sample size. Furthermore, DHF is beneficial for sepsis, the incidence of which was higher in the DHF group, and this might have improved the overall outcome.

Our study has several limitations. It was a single-center study with a small sample size, and the time of the survey period may have had some influence on the result, due to changes in surgery procedures and other clinical treatments, but our in-hospital mortality rate was comparable to the previously published rates of 38.8% - 59% for cardiac surgery RTT patients [8,9,11,26].

## Conclusions

Our results suggest that GDRRT is safe and effective for patients with AKI after cardiac surgery and is superior to DHF for improving renal recovery. In addition, it reduces hospitalization time and expenses. GDRRT can be applied to most AKI patients after cardiac surgery, while DHF may be more suitable for critically ill AKI patients with sepsis or MODS.

## Abbreviations

GDT: Goal directed therapy; EGDT: Early goal-directed fluid therapy; RRT: Renal replacement therapy; GDRRT: Goal-directed renal replacement therapy; DHF: Daily hemofiltration; HD: Hemodialysis; HF: Hemofiltration; HDF: Hemodiafiltration; AKI: Acute kidney injury; CRRT: Continuous renal replacement therapy; IRRT: Intermittent renal replacement therapy; SLED: Sustained low-efficiency dialysis; ICU: Intensive care unit; APACHE: Acute physiology and chronic health evaluation; SOFA: Sepsis-related organ failure assessment; BUN: Blood urea nitrogen; SCr: Serum creatinine; MAP: Mean arterial pressure; CVP: Central venous pressure; LMWH: Low-molecular-weight heparin; ARDS: Acute respiratory distress syndrome; MODS: Multiple organ dysfunction syndrome; CABG: Coronary artery bypass graft; CVVH: Continued veno-veno hemofiltration; ESRD: End-stage renal disease.

## Competing interests

All authors declare that they have no competing interests.

## Authors’ contributions

JRX developed the study protocol, collected and analyzed the data, and wrote manuscript. XQD designed the study and provided critical review of the manuscript. YF provided critical review of the manuscript. BS and ZHL collected some of the data. JZZ designed the study and provided critical review of the manuscript. LL and CSW collected some of the data. JT conceived the study, developed the study protocol and provided critical review of the manuscript. All authors read and approved the final manuscript.
